# Redox interplay between mitochondria and peroxisomes

**DOI:** 10.3389/fcell.2015.00035

**Published:** 2015-05-27

**Authors:** Celien Lismont, Marcus Nordgren, Paul P. Van Veldhoven, Marc Fransen

**Affiliations:** Laboratory of Lipid Biochemistry and Protein Interactions, Department of Cellular and Molecular Medicine, KU Leuven – University of LeuvenLeuven, Belgium

**Keywords:** antioxidant systems, interorganellar cross-talk, mitochondria, oxidative stress, peroxisomes, pro-oxidant systems, redox signaling

## Abstract

Reduction-oxidation or “redox” reactions are an integral part of a broad range of cellular processes such as gene expression, energy metabolism, protein import and folding, and autophagy. As many of these processes are intimately linked with cell fate decisions, transient or chronic changes in cellular redox equilibrium are likely to contribute to the initiation and progression of a plethora of human diseases. Since a long time, it is known that mitochondria are major players in redox regulation and signaling. More recently, it has become clear that also peroxisomes have the capacity to impact redox-linked physiological processes. To serve this function, peroxisomes cooperate with other organelles, including mitochondria. This review provides a comprehensive picture of what is currently known about the redox interplay between mitochondria and peroxisomes in mammals. We first outline the pro- and antioxidant systems of both organelles and how they may function as redox signaling nodes. Next, we critically review and discuss emerging evidence that peroxisomes and mitochondria share an intricate redox-sensitive relationship and cooperate in cell fate decisions. Key issues include possible physiological roles, messengers, and mechanisms. We also provide examples of how data mining of publicly-available datasets from “omics” technologies can be a powerful means to gain additional insights into potential redox signaling pathways between peroxisomes and mitochondria. Finally, we highlight the need for more studies that seek to clarify the mechanisms of how mitochondria may act as dynamic receivers, integrators, and transmitters of peroxisome-derived mediators of oxidative stress. The outcome of such studies may open up exciting new avenues for the community of researchers working on cellular responses to organelle-derived oxidative stress, a research field in which the role of peroxisomes is currently highly underestimated and an issue of discussion.

## Introduction

**Figure 1:**
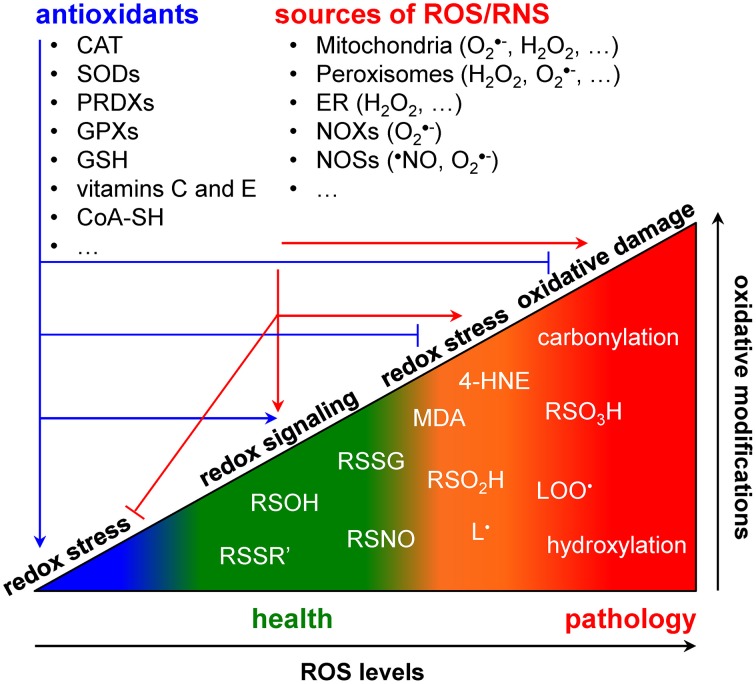
**ROS/RNS, antioxidants, and cellular redox balance in health and disease**.

All life on earth is powered by reduction-oxidation (redox) reactions, in which electrons are transferred from a donor to an acceptor molecule. To survive, cells had to evolve mechanisms to control redox potential intervals in which biological processes and signaling pathways can take place (Foyer and Noctor, 2011). To cope with this challenge, cells have developed spatially compartmentalized redox circuits that are regulated by various small molecule- and protein-based redox buffer systems (Forman et al., 2010; Mallikarjun et al., 2012). As organisms need to sense and respond to changing environmental conditions, these circuits have to be sufficiently flexible to (locally) respond to exogenous stimuli and endogenous metabolic alterations. Shifts in the intracellular redox equilibrium may favor either beneficial or detrimental outcomes (Figure 1). The outcomes are determined by a combination of factors, including the types of oxidants produced, their concentration and localization, and their kinetics of production and elimination (Trachootham et al., 2008; Forman et al., 2010). A shift of the redox equilibrium in favor of oxidized biomolecules gives rise to a phenomenon called “oxidative stress.” High levels of oxidative stress are capable of causing damage to all major biomolecules and of initiating cell death (Jones and Go, 2010). However, low levels of oxidative stress may promote cell proliferation and survival pathways (Holmström and Finkel, 2014). As such, it is not surprising to see that changes in the cellular redox environment significantly contribute to the development of virtually all major chronic human disorders, including atherosclerosis, cancer, diabetes, and neurodegeneration (Groitl and Jakob, 2014; Holmström and Finkel, 2014).

Redox signaling refers to the concept that electron-transfer processes play a key messenger role in biological systems (Rigas and Sun, 2008; Burgoyne et al., 2012). Cells produce two different types of redox signaling molecules: the first type comprises reactive oxygen species (ROS), such as the superoxide anion radical (O^•−^_2_), hydrogen peroxide (H_2_O_2_), and the hydroxyl radical (^•^OH); the second type covers reactive nitrogen species (RNS), such as the nitric oxide radical (^•^NO), the nitrogen dioxide radical (^•^NO_2_), nitrite (NO^−^_2_), and peroxynitrite (ONOO^−^) (Nathan and Ding, 2010). Oxidative modifications of specific target molecules by various ROS/RNS are covalent but often reversible. The best studied reversible oxidation reactions include those of H_2_O_2_ with sulfhydryl groups (RSH) to yield disulfides (RSSR'), sulfenic acids (RSOH), or sulfinic acids (RSO_2_H), and of ^•^NO with RSH to yield S-nitrosothiols (RSNO) (Nathan and Ding, 2010). Irreversible oxidation products most frequently include hydroxylations, carbonylations, nitrations, the formation of sulfonic acids (RSO_3_H), and the destruction of iron-sulfur (FeS) clusters (Nathan and Ding, 2010). As both protein cysteine thiols and lipids are among the most prominent targets of ROS/RNS (Trachootham et al., 2008; Hekimi et al., 2011), many biologically-relevant redox signals are conveyed through cysteine oxidation and lipid peroxidation. Here, it is of particular importance to be aware that (i) many signaling components like kinases, phosphatases, transcription factors, caspases, and metalloproteases contain active site- or zinc finger-coordinating cysteines that can be reversibly modified in a redox-responsive manner (Forman et al., 2010; Corcoran and Cotter, 2013; Berridge, 2014), and (ii) multiple lipid peroxidation products [e.g., malondialdehyde (MDA) and 4-hydroxy-2-nonenal (4-HNE)] can act as important messengers in signaling events that lead to cell proliferation, differentiation, senescence, or apoptosis (Fritz and Petersen, 2013; Ayala et al., 2014). Finally, to counteract oxidative stress, cells are also equipped with various antioxidant defense systems. These systems can be classified into two broad categories: the enzymatic antioxidants [e.g., superoxide dismutases (SODs), catalase, glutathione peroxidases and reductases, peroxiredoxins, etc.) and the low molecular weight antioxidants [e.g., reduced glutathione (GSH), ascorbate (vitamine C), α-tocopherol (vitamin E), etc.] (Nathan and Ding, 2010).

Major sites of cellular ROS/RNS production include mitochondria, peroxisomes, the endoplasmic reticulum (ER), and the NADPH oxidases (NOXs) and nitric oxide synthases (NOSs) that are located in distinct subcellular locations (Trachootham et al., 2008; Fransen et al., 2012). In the following two sections, we will review the pro- and antioxidant systems of mitochondria and peroxisomes, with a focus on the situation in mammals. For a detailed description of the other systems, the reader is referred to other reviews (Bedard and Krause, 2007; Appenzeller-Herzog, 2011; Förstermann and Sessa, 2012).

## The redox metabolism of mitochondria

### Pro-oxidant systems

Mitochondria are key players in cellular redox metabolism. Important sources of mitochondrial ROS include (i) complex I and complex III of the electron transport chain (ETC) in the inner mitochondrial membrane, (ii) dihydrolipoamide dehydrogenase (DLD), a common subunit of the α-ketoglutarate dehydrogenase and pyruvate dehydrogenase multi-enzyme complexes, in the mitochondrial matrix (Andreyev et al., 2005; Circu and Aw, 2010), and (iii) monoamine oxidase, a mitochondrial outer membrane-resident flavoprotein (Andreyev et al., 2005; Orrenius et al., 2007). Monoamine oxidase is an important source of H_2_O_2_ (Orrenius et al., 2007). Complex I, complex III, and dihydrolipoamide dehydrogenase contain redox centers that are potentially capable of O^•−^_2_ production (Andreyev et al., 2005). Importantly, while complex I can only produce O^•−^_2_ in the mitochondrial matrix, complex III can release this radical on both sides of the mitochondrial inner membrane (Orrenius et al., 2007). Excessive production of O^•−^_2_ may not only lead to the SOD-catalyzed formation of H_2_O_2_, but also cause the release of Fe^2+^ from FeS-containing proteins (e.g., complex I, aconitase) (Dixon and Stockwell, 2014). These events may in turn give rise to ^•^OH via the non-enzymatic Haber-Weiss and Fenton reactions (Winterbourn, 1995) and initiate a cascade of reactions resulting in the formation of carbon-centered lipid radicals (L^•^), lipid peroxide radicals (LOO^•^), and multiple lipid peroxidation products such as MDA and 4-HNE (Ayala et al., 2014). The role of mitochondria in cellular RNS production is less well documented. However, here it is worthwhile to mention that the mitochondrial inner membrane contains at least three NO^•^-producing enzymes: a posttranslationally modified splice variant of neuronal NOS (NOS1α), called mitochondrial NOS (mtNOS) (Ghafourifar and Richter, 1997; Aguirre et al., 2012); and two molybdopterin-containing amidoxime-reducing enzymes, called MARC1 and MARC2 (Sparacino-Watkins et al., 2014). Wheareas, in general, NOSs oxidize L-arginine with O_2_ to form citrulline and ^•^NO, molybdopterin enzymes have the capacity to reduce NO^−^_2_ to ^•^NO (Sparacino-Watkins et al., 2014).

### Antioxidant systems

Mitochondria also contain a network of enzymatic and non-enzymatic antioxidants that protect the organelle from oxidative damage. The main antioxidant enzymes include thioredoxin 2 (TRX2), thioredoxin reductase (TXNRD2), the glutaredoxins 2 (GLRX2) and 5 (GLRX5), the peroxiredoxins 3 (PRDX3) and 5 (PRDX5), GSH peroxidase 1 (GPX1), oxidized glutathione (GSSG) reductase (GSR), and the copper/zinc (SOD1)- and manganese (SOD2)-containing SODs (Kalinina et al., 2008). The major non-enzymatic antioxidants are GSH, coenzyme A (CoA-SH; for more details, see The Redox Metabolism of Peroxisomes - Antioxidant Systems), ubiquinol (Maroz et al., 2009), vitamin C, and vitamin E (Sagun et al., 2005; Marí et al., 2009; Lauridsen and Jensen, 2012). Vitamin E, a fat-soluble nutrient, is present in relatively low concentrations in mitochondria, and its main function is to trap LOO^•^, thereby preventing the propagation of lipid peroxidation (Forkink et al., 2010). GSH and vitamin C, two hydrophilic antioxidants, can directly recycle vitamin E to its reduced active form. GSH is the most important low molecular weight thiol (~1-6 μmol/g tissue), and its concentration in mitochondria is estimated at ~10–14 mM, similar to the cytosolic levels. GSH can also directly neutralize ^•^OH and function as a cofactor of GPX1 to scavenge H_2_O_2_ and lipid peroxides (LOOH) (Forkink et al., 2010). The regeneration of GSH from GSSG is carried out by GSR, an NADPH-consuming enzyme. Note that, as GSH is only synthesized *de novo* in the cytosol, mitochondria have to import this molecule across their inner membrane (Marí et al., 2009). An alternative mechanism to convert mitochondrial H_2_O_2_ to H_2_O involves the oxidation of PRDX3 or PRDX5. The oxidized forms of these peroxiredoxins are subsequently reduced by TRX2, which in turn is regenerated by TXNRD2, an NADPH-dependent FAD-containing enzyme (Forkink et al., 2010). Two comments should be added at this point. First, the GSH/GPX/GLRX and PRDX/TRX/TXNRD redox pathways are considered to be the most important redox regulating systems in mitochondria (Murphy, 2012). Second, as (i) GSR and TXNRD2 receive their reducing equivalents from the mitochondrial NADPH pool, and (ii) the pool of NADPH is kept reduced by the tricarboxylic acid (TCA) enzymes nicotinamide nucleotide transhydrogenase (NNT), malic enzyme 3 (ME3), and isocitrate dehydrogenase 2 (IDH2), it is clear that a functional TCA cycle is essential for the regeneration of the antioxidant capacity of the mitochondrial matrix (Kohlhaas and Maack, 2013).

### Metabolic factors affecting ROS production

The net release of ROS from mitochondria strongly depends on the (patho) physiological state of the cell. For example, according to the classical concept, the rate of ROS production from the ETC increases when substrates (e.g., glucose, fatty acids) are available, but energy consumption is low (Kohlhaas and Maack, 2013). Indeed, when TCA-derived high-energy electron carriers NADH and FADH2 donate more electrons to the ETC and mitochondria are not making ATP, the ETC is highly reduced and electrons are more likely to slip to O_2_ to produce O^•−^_2_ (Kohlhaas and Maack, 2013). In addition, also stress situations causing impairment and uncoupling of specific respiratory chain complexes (RCCs) may provoke the formation of free radicals (Schönfeld and Wojtczak, 2008; Marí et al., 2009).

## The redox metabolism of peroxisomes

### Pro-oxidant systems

As peroxisomes contain large sets of pro- and antioxidant enzymes, also these organelles have the potential to play a significant role in cellular redox metabolism and signaling. The most abundant class of ROS-producing enzymes inside peroxisomes are the flavin-containing oxidases, which reduce O_2_ to H_2_O_2_ [for a detailed overview of these enzymes, the reader is referred to (Antonenkov et al., 2010)]. Depending on the organism, the tissue and cell type, and the cellular environment, peroxisomes also contain two potential sources of O^•−^_2_ and ^•^NO production: xanthine dehydrogenase (XDH) and the inducible form of NOS (NOS2) (Angermüller et al., 1987; Stolz et al., 2002; Loughran et al., 2013). XDH is a key enzyme in the purine degradation pathway that catalyzes the conversion of hypoxanthine to xanthine and of xanthine to uric acid, a potent antioxidant and free radical scavenger (Nishino et al., 2008). However, a select set of posttranslational modifications (e.g., sulfhydryl oxidation, proteolytic processing) can rapidly convert the NAD^+^-dependent dehydrogenase form of the enzyme to an oxidase form that catalyzes the reduction of O_2_ to O^•−^_2_ (Nishino et al., 2008). As XDH, like MARC1 and MARC2 (see The Redox metabolism of Mitochondria - Pro-oxidant Systems), is a molybdopterin-containing enzyme, it can also reduce nitrates and NO^−^_2_ to NO^•^ (Harrison, 2002). NOS2 is a homodimeric heme-containing enzyme that normally catalyzes the oxidation of L-arginine to NO^•^ and citrulline in a complex reaction requiring O_2_, NADPH, tetrahydrobiopterin (BH4), FMN, and FAD (Del Río, 2011). However, in the absence of substrate or in its monomeric form, the enzyme can also produce significant amounts of O^•−^_2_ (Stuehr et al., 2001). Interestingly, the peroxisomal pool of NOS2 appears to be monomeric (Loughran et al., 2005). Finally, as (i) NO^•^ may rapidly combine with O^•−^_2_ to form ONOO^−^ (Pacher et al., 2007), (ii) within the heme protein-rich environment of peroxisomes, H_2_O_2_ may give rise to ^•^OH through the Fenton reaction (Loughran et al., 2005), and (iii) ^•^OH is one of the prime catalysts for the initiation of lipid peroxidation (Ayala et al., 2014), it is very likely that peroxisomes also function as potential sources of ONOO^−^, ^•^OH, L^•^, LOO^•^, MDA, and 4-HNE (Ayala et al., 2014).

### Antioxidant systems

Like mitochondria, peroxisomes are also well equipped with multiple enzymatic and non-enzymatic antioxidant defense systems that scavenge harmful H_2_O_2_ and free radicals, thereby protecting the organelle from oxidative stress. The best characterized peroxisomal antioxidant enzyme is catalase, a heme-containing enzyme that can remove H_2_O_2_ in a catalatic (2 H_2_O_2_ → 2 H_2_O + O_2_) and peroxidatic (H_2_O_2_ + AH_2_ → A + 2 H_2_O) manner (Kirkman and Gaetani, 2007). Typical peroxidatic electron donors (AH_2_) are low molecular weight alcohols, formate, nitrite, and formaldehyde (Kirkman and Gaetani, 2007). Other antioxidant enzymes include SOD1, PRDX5, glutathione transferase kappa (GSTK1), “microsomal” glutathione S-transferase 1 (MGST1), and epoxide hydrolase 2 (EPHX2). As already mentioned above, SOD1 can convert O^•−^_2_ to O_2_ and H_2_O_2_ (see The Redox metabolism of Mitochondria - Pro-oxidant Systems), and PRDX5 can reduce H_2_O_2_ to H_2_O (see The Redox metabolism of Mitochondria - Antioxidant Systems). PRDX5 can, in addition, also reduce alkyl hydroperoxides (ROOH) to their respective alcohols, and ONOO^−^ to NO^−^_2_ (Knoops et al., 2011). GSTK1 and MGST1 are thought to play a role in LOOH detoxification processes (Antonenkov et al., 2010; Johansson et al., 2010; Wang et al., 2013b), EPHX2 can convert epoxides to the corresponding dihydrodiols (Decker et al., 2009), and peroxisomal PRDX5 has recently been shown to exert a cytoprotective function against H_2_O_2_- and LOOH-induced oxidative stress (Walbrecq et al., 2015). For a detailed description of these enzymes, the reader is referred to other reviews (Antonenkov et al., 2010; Fransen et al., 2012). Interestingly, there is some indirect evidence that also GSH and vitamin C may play a role in the regulation of the peroxisomal redox state. Indeed, as the peroxisomal membrane contains a nonselective pore-forming protein (PXMP2) with an upper molecular size limit of 300-600 Da (Rokka et al., 2009), these low molecular weight antioxidants can most likely freely diffuse through the peroxisomal membrane. The observations that (i) a peroxisomal variant of roGFP2, a genetically-encoded redox sensor that specifically equilibrates with the GSSG/GSH redox pair, quickly responds to redox changes in the peroxisomal matrix, and (ii) supplementation of vitamin C to the cell culture medium causes an increase in the intraperoxisomal redox state, are in line with this hypothesis (Ivashchenko et al., 2011). However, the precise mechanisms underlying the latter, rather unexpected observation remain to be unraveled. Nevertheless, in the context of this review, it is tempting to speculate that, in a heme-rich environment such as the peroxisomal matrix, vitamin C can reduce Fe^3+^ to Fe^2+^, and that this further drives the generation of free radicals through the Fenton reaction. Note also that it is not yet clear how GSSG (molar mass, 612.63 g mol^−1^) can be reduced in or exported out of the peroxisomal matrix. Here it is important to point out that, in contrast to mitochondria (see The Redox metabolism of Mitochondria - Antioxidant Systems), peroxisomal thioredoxins and glutaredoxins have not yet been identified in mammals. Finally, it should be noted that—although peroxisomes contain (i) enzymes that can produce and consume NAD(P)^+^ and NAD(P)H (Visser et al., 2007), and (ii) shuttle systems that permit the transfer of reducing equivalents without physical exchange of these redox cofactors between the lumen of the organelle and the cytoplasm (Rottensteiner and Theodoulou, 2006; Antonenkov et al., 2010; Schueren et al., 2014)—it is also not yet clear how changes in peroxisomal NAD(P)^+^ and NAD(P)H metabolism influence the intra- and extraperoxisomal redox state.

### Metabolic factors affecting ROS production

So far, no consensus has been reached about whether peroxisomes function as a net source or sink of ROS/RNS (Fransen et al., 2013). However, as for mitochondria, this most likely depends on the (patho) physiological state and growth environment of the cell. This idea is in line with the observation that the intraperoxisomal redox state is strongly influenced by various genetic and environmental factors (Ivashchenko et al., 2011). In the following subsections, we further discuss how peroxisomal metabolism of fatty acids, acyl-CoA esters, and plasmalogens may impact on cellular redox state alterations. Not unexpectedly, most of these lipids are linked to two of the major metabolic pathways in peroxisomes: β-oxidation and plasmalogen synthesis.

#### Fatty acids and acyl-CoA esters

Mammalian genomes code for at least three functional peroxisomal acyl-CoA oxidases (ACOX1, ACOX2, ACOX3), and evidence exists for a fourth gene (*ACOXL*) (Van Veldhoven, 2010). Additionally, the *ACOX1* gene gives rise to two transcripts via alternative splicing, and this splicing is conserved in eukaryotes (Morais et al., 2007). Through these different ACOXs, combined with an uptake mechanism for CoA-esters that is not controlled and restricted by a carnitine-acylcarnitine translocase as in mitochondria (Rubio-Gozalbo et al., 2004), peroxisomes can β-oxidize a broad range of carboxylates, including medium-, long-, and very-long-chain fatty acids, mono- and polyunsaturated fatty acids, pristanic acid and other 2-methyl-branched fatty acids, as well as carboxylates with a bulky or rigid ω-end such as prostanoids, bile acid intermediates and xenobiotics, either with or without an α-methyl group (Van Veldhoven, 2010). Moreover, these organelles can degrade carboxylates containing a 3-methyl or 2-hydroxy group via α-oxidation (Van Veldhoven, 2010), whereby the one-carbon shortened products can be passed onto the β-oxidation system. Exposure of cells to carboxylates (or their precursors) that are desaturated by these ACOXs will generate peroxisomal H_2_O_2_. This has been shown in different systems [e.g., cells, perfused organs (Foerster et al., 1981; Handler and Thurman, 1987), and intact animals (Van den Branden et al., 1984)] and with different types of carboxylates [e.g., medium-chain fatty acids (Skorin et al., 1992); long-chain saturated and mono- and polyunsaturated fatty acids (Mannaerts et al., 1979; Foerster et al., 1981; Chu et al., 1995; Okamoto et al., 1997)]; medium-chain dicarboxylic acids (Leighton et al., 1986); and xenobiotics such as *N*-(*α*-methylbenzyl)azelaamic acid (Yamada et al., 1986; Suzuki et al., 1990), ω-phenyl-substituted fatty acids (Yamada et al., 1987), and PCA16, a metabolite of the cytosine arabinoside antileukemic prodrug YNKO (containing a stearic acid side chain) (Yoshida et al., 1990).

The amount of H_2_O_2_ produced depends strongly on the chain length of the fatty acids. For example, when comparing fatty acids ranging from C8:0 to C18:0 in isolated rat hepatocytes, a maximal activity is observed around C10:0-C12:0 (Yamada et al., 1987; Suzuki et al., 1990; Skorin et al., 1992). Similarly, ω-phenyldodecanoic acid (Yamada et al., 1987) and dodecanedioic acid (Leighton et al., 1989) are optimal compared to their analogs. Finally, monounsaturated oleic acid is a better H_2_O_2_-source than palmitic acid in rat hepatocytes (Mannaerts et al., 1979) and perfused rat liver (Foerster et al., 1981). In mice, starvation increases the H_2_O_2_ production by liver, likely due to increased fatty acid plasma levels (Van den Branden et al., 1984). However, in cardiac tissue there is no change (Kerckaert and Roels, 1986). Treatment with fibrates increases the rate of H_2_O_2_ production in hepatocytes (Mannaerts et al., 1979; Foerster et al., 1981; Yamada et al., 1987; Leighton et al., 1989; Yoshida et al., 1990).

For various fatty acids linked to peroxisomal metabolism, an influence on ROS levels has been reported. Examples include (i) phytanic acid (3,7,11,15-tetramethylhexadecanoic acid), a dietary fatty acid that is degraded via peroxisomal α-oxidation, (ii) pristanic acid (2,6,10,14-tetramethylpentadecanoic acid), the breakdown product of phytanic acid, that is degraded via β-oxidation, (iii) polyunsaturated fatty acids (PUFAs) that are produced via the retroconversion pathway, and (iv) VLCFAs that are shortened via β-oxidation (Van Veldhoven, 2010). Interestingly, long-chain fatty acids that are normally degraded by mitochondria have been shown to be toxic to insulin-producing cells (e.g., RINm5F, INS-1E, and primary rat islet cells), due to the combined effect of increased peroxisomal H_2_O_2_ generation and the intrinsically low activity of catalase in these cells (Gehrmann et al., 2010).

#### Plasmalogens

An important class of ROS-protecting lipids are plasmalogens. This class of phospholipids contains a vinyl ether bond at position one of the glycerol moiety. The initial steps of their biosynthesis are confined to peroxisomes: glyceronephosphate O-acyltransferase (GNPAT) acylates dihydroxyacetone-phosphate, while alkylglycerone-phosphate synthase (AGPS) replaces the acylgroup by a fatty alcohol (Braverman and Moser, 2012). Subsequently, the 2-oxogroup is reduced by 1-alkylglycerone-phosphate reductase (DHRS7B), an enzyme found in the membrane of both peroxisomes and the ER (Keller et al., 2009; Lodhi et al., 2012), thereby generating 1-alkylglycero-3-phosphate which will undergo further metabolic conversions in the ER, similar to those of 1-acylglycero-3-phosphate [the precursor of esterglycero(phospho)lipids]. Finally, the double bond adjacent to the ether bond is introduced in the ER (Nagan and Zoeller, 2001). At the sn-2 position, plasmalogens are generally enriched in PUFAs. In some tissues (e.g., brain, testis, and heart), a substantial portion (10–30%) of the phospholipids (especially the ethanolamine-phospholipids) are plasmalogens. The absence of plasmalogens causes a very specific phenotype in man, described as rhizomelic chondrodysplasia punctata (dwarfism, shortening of proximal limbs, etc.), but how these symptoms are linked to plasmalogens is not clear (Braverman and Moser, 2012).

The vinyl-ether bond makes plasmalogens sensitive to attack by different ROS-species, both *in vitro* and *in cellulo*. Hence, one can consider them as (lipophilic) antioxidants (Lessig and Fuchs, 2009), often comparable to tocopherol with regard to potency. *In vitro*, plasmalogens delay the oxidative degradation of PUFAs as good as vitamin E (Engelmann et al., 1994; Reiss et al., 1997; Hahnel et al., 1999a), most likely due to the fact that the vinyl-ether bond can scavenge peroxy radicals and oxidized PUFA products. As this bond can complex with Cu^2+^, plasmalogens also attenuate Cu^2+^-induced lipid oxidation (Hahnel et al., 1999b). Depending on the type of oxidative stress, different metabolites can be formed. For example, while UV light-induced oxidation of plasmalogens generates aldehydes via dioxetane intermediates, Fe^2+^/ascorbate treatment results in the formation of α-hydroxy-aldehydes via plasmalogen epoxides (Stadelmann-Ingrand et al., 2001). Plasmalogens do protect cells against chemical hypoxia induced by antimycin A or cyanide (by scavenging produced ROS), as shown in the murine monocyte/macrophage cell line RAW 264.7 (Zoeller et al., 1999) and human pulmonary arterial endothelial cells (PAEC) (Zoeller et al., 2002). During UV-exposure of Chinese hamster ovary (CHO) cells photosensitized with ω-pyrene-substituted fatty acids or Merocyanine 540, plasmalogens disappear (Zoeller et al., 1988). This is most likely due to the fact that singlet oxygen converts the vinyl-ether bond into a dioxetane intermediate that subsequently decomposes into a 2-lysophospholipid, formic acid, and an n-1 aldehyde (Zoeller et al., 1988). Another ROS-molecule that is scavenged by plasmalogens is hypochlorous acid (HOCl). This reactive chlorinating species is produced by myeloperoxidase and promotes the selective cleavage of plasmalogens into 1-lysophosphatidylcholine and 2-chloro-fatty aldehydes. This has been shown *in vitro* (Albert et al., 2001; Messner et al., 2006; Skaff et al., 2008; Ullen et al., 2010), in activated neutrophils (Thukkani et al., 2002) and monocytes (Thukkani et al., 2003), and in mouse brain with lipopolysacharide-induced neuroinflammation (Ullen et al., 2010). Likewise, 2-bromo-fatty aldehydes are produced by stimulated neutrophils (Albert et al., 2002) or eosinophils (Albert et al., 2003). Note that 2-halo-fatty aldehydes, being chemoattractants for phagocytes and stimulators of expression of phagocyte tethering proteins in endothelial cells, sustain the inflammatory response. Finally, in thyroid cells, plasmalogens are susceptible to iodine attack. This results in the formation of 2-iodo-fatty aldehydes (Panneels et al., 1996), a major thyroid iodolipid that—similar to iodide—regulates thyroid metabolism. Cells with higher plasmalogen levels are more resistant to H_2_O_2_, hyperoxia, and the O^•−^_2_ generator plumbagin (Zoeller et al., 2002), whereas the protective actions are gone in cells lacking plasmalogens. Photosensitisized plasmalogen-deficient CHO cells and mouse embryonic fibroblasts are hypersensitive to light treatment (Zoeller et al., 1988; Wang et al., 2013b), and plasmalogen-deficient RAW 264.7 cells are more sensitive to electron transport inhibitors (Zoeller et al., 1999). However, plasmalogens are apparently not important to protect cells against lactic acid-induced oxidative stress, at least not in primary rat astrocytes (Fauconneau et al., 2001).

To conclude this subsection, it should be noted that plasmalogen-derived oxidation products, and especially 2-hydroxy-fatty aldehydes (Liu and Sayre, 2003; Stadelmann-Ingrand et al., 2004) and 2-halo-fatty aldehydes (Stadelmann-Ingrand et al., 2004; Wildsmith et al., 2006), are reactive molecules that can modify amino groups of lipids and proteins (via Schiff base formation) as well as sulfhydryl groups in proteins. This probably contributes to their short half-life in cerebral cortex homogenates (Stadelmann-Ingrand et al., 2001). In addition, oxidation of 2-chloro-fatty aldehydes to 2-chloro-fatty acids leads to increased ROS, ER-stress, and finally apoptosis in activated primary human monocytes, THP-1 human monocytes, and RAW 264.7 mouse macrophages (Wang et al., 2013a). Together with the observations that (i) α-hydroxy fatty aldehydes and plasmalogen epoxides accumulate in aged brain and chronic disorders (Lessig and Fuchs, 2009), and (ii) 2-chloroaldehyde levels are elevated in atherosclerotic plaques and infarcted rat myocardium (Ford, 2010), these findings question the scavenger role of plasmalogens. However, it might be that the pathways degrading these oxidative metabolites are less active under these conditions.

## Mitochondria as redox signaling nodes

**Figure 2:**
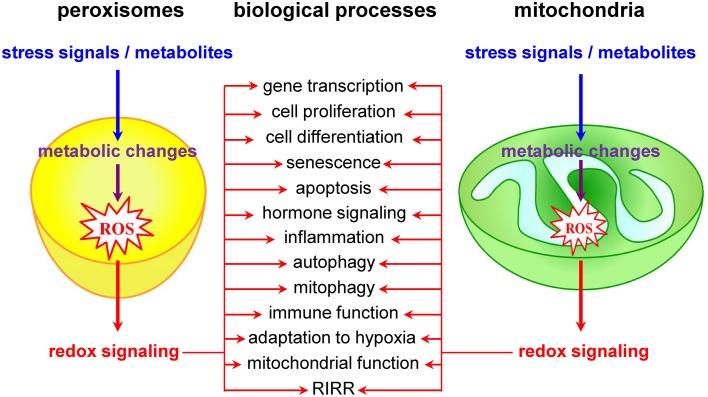
**Peroxisomes and mitochondria actively contribute to transcompartmental redox signaling**.

Currently, it is widely accepted that redox signals to and from mitochondria are at the core of a wide variety of biological processes, including cell proliferation and differentiation, adaptation to hypoxia, autophagy, immune function, and hormone signaling (Figure 2) (Collins et al., 2012; Chandel, 2014). The most studied and best characterized mitochondrial redox signaling molecule is H_2_O_2_, which is relatively stable *in vivo* and can pass easily through mitochondrial membranes (Bienert et al., 2006). For example, it has been shown that, under physiological hypoxia, mitochondrial H_2_O_2_ can stabilize hypoxia-inducible factor 1α (HIF-1α), a transcription factor playing a key role in the cellular adaptation to oxygen availability (Chandel et al., 1998). In addition, also other transcription factors (e.g., FOXO3A, NF-kB, p53, and PGC-1α) and signaling components (e.g., c-Jun N-terminal kinase, protein tyrosine phosphatases, cysteine protease Atg4, the mitochondrial peroxiredoxins, the NLRP3 inflammasome, etc.) have been identified as targets of mitochondrial H_2_O_2_ (Chandel et al., 2000a,b; Nemoto et al., 2000; Valle et al., 2005; Scherz-Shouval et al., 2007; Chiribau et al., 2008; Cox et al., 2010; Zhou et al., 2011; Chae et al., 2013; Frijhoff et al., 2014; Long et al., 2014; Marinho et al., 2014). However, although it is well known that H_2_O_2_ can selectively modify proteins containing cysteine residues with a low pKa (Veal et al., 2007), the precise mechanisms by which mitochondria-derived H_2_O_2_ coordinates or relays (retrograde) signaling events thereby provoking adaptive or maladaptive responses are not yet entirely clear (Forkink et al., 2010). Two main models have been proposed: (i) in the redox relay model, H_2_O_2_ scavenging enzymes are first oxidized and subsequently transfer the oxidative equivalents to other target proteins (Toledano et al., 2004); and (ii) in the floodgate model, scavenging enzymes act as molecular floodgates, keeping H_2_O_2_ away from susceptible targets under basal conditions, but permitting signaling events to occur at H_2_O_2_ thresholds sufficient to inactivate the scavenging enzymes (Wood et al., 2003).

Another important class of redox signaling molecules are cardiolipins, which are virtually exclusively localized in the inner mitochondrial membrane (Ren et al., 2014). Most cardiolipin species have four unsaturated acyl chains that are oxidation-sensitive. Currently, it is well documented that—upon mitochondrial injury and depolarization—a significant portion of these lipids is externalized to the mitochondrial surface where they function as an “eat-me”-signal for the autophagic machinery (Chu et al., 2013). In addition, detailed studies have established that cardiolipin peroxidation is a critical first step in cytochrome c release and apoptosis (Kagan et al., 2014). The precise mechanisms through which peroxidized cardiolipin species fulfill their action remain to be established, but most likely they facilitate outer mitochondrial membrane permeabilization (Kagan et al., 2014; Raemy and Martinou, 2014).

Over the years, it has become clear that mitochondria are central integrators and transducers for ROS signals from other cellular sources (Sena and Chandel, 2012). In this context, it is interesting to briefly discuss the concept of ROS-induced ROS release (RIRR), a phenomenon wherein mitochondria respond to elevated ROS concentrations by increasing their own ROS production (Zorov et al., 2000). RIRR is a mechanism for ROS amplification and regional ROS generation. The process involves the opening of two different channels in the mitochondrial inner membrane: the mitochondrial permeability transition pore (mPTP) and the inner membrane anion channel (IMAC) (Aon et al., 2009; Zorov et al., 2014). Opening of the pores (e.g., upon elevated Ca^2+^ or ROS levels) may, among other responses [e.g., a dissipation of the mitochondrial inner membrane potential (Δ Ψ m), a ceased production of ATP, etc.], elicit ROS bursts. Depending on the extent of pore opening and how fast ROS released from mitochondria are eliminated by intracellular antioxidant systems, RIRR may (i) constitute an adaptive housekeeping mechanism to release accumulated toxic levels of mitochondrial ROS, (ii) activate pools of redox-sensitive proteins in the vicinity of mitochondria, (iii) trigger RIRR in neighboring mitochondria, and (iv) lead to the destruction of mitochondria, and—if propagated from mitochondrion to mitochondrion—of the cell itself (Zinkevich and Gutterman, 2011; Zorov et al., 2014).

## Peroxisomes as redox signaling nodes

Although it is already known for years that peroxisomal metabolism and cellular redox equilibrium are closely intertwined (Reddy and Rao, 1987), peroxisomes have long been underestimated and largely ignored as potential redox signaling platforms. However, a limited but growing number of studies lend strong support to the idea that these organelles do actively contribute to transcompartmental ROS signaling in mammalian cells (Figure 2). For example, alterations in peroxisomal H_2_O_2_ metabolism have been shown to influence the cellular protein disulfide content (Yang et al., 2007; Ivashchenko et al., 2011), NF-kB activation (Li et al., 2000; Han et al., 2014), E-cadherin expression (Han et al., 2014), the secretion of matrix metalloproteinases (Koepke et al., 2008; Han et al., 2014), mTORC1 activity and autophagy (Zhang et al., 2013), neuronal activity (Diano et al., 2011), and cell fate decisions in response to different stressors (Carter et al., 2004; Chen et al., 2004; Elsner et al., 2011). Unfortunately, the precise molecular mechanisms underlying most of these observations remain poorly understood and sometimes even controversial. For example, although these and other observations strongly indicate that H_2_O_2_ can rapidly cross the peroxisomal membrane (Boveris et al., 1972; Fritz et al., 2007), the molecular identity of the channels involved remains to be determined. In addition, virtually nothing is known about how peroxisome-derived H_2_O_2_ can coordinate or relay signaling events. In this context, it is important to highlight and briefly discuss one of the potentially most significant recent breakthroughs in this research area. It concerns the discovery that the tuberous sclerosis complex (TSC) signaling node (TSC1, TSC2, TBC1D7, and Rheb) can localize to peroxisomes, and that this localization is essential to regulate mTORC1 activity in response to (peroxisomal) ROS (Zhang et al., 2013). However, as (i) the peroxisomal localization of TSC2 could not yet be confirmed by others (Menon et al., 2014), and (ii) peroxisome proliferator-activated receptor (PPAR) agonist-induced ROS production and exogenous H_2_O_2_ addition do not faithfully mimic the spatial and temporal signaling pattern of peroxisome-derived H_2_O_2_ [e.g., PPAR agonists increase ROS production by both peroxisomal and non-peroxisomal enzymes (Pyper et al., 2010)], these findings should be interpreted with care and warrant future research. In the context of this section, it is also worth mentioning that, albeit the intraperoxisomal redox status is strongly influenced by environmental growth conditions, peroxisomes resist—within limits—oxidative stress generated elsewhere in the cell (Ivashchenko et al., 2011). However, as an increase in the redox state of the cytosol reduces the import efficiency of peroxisomal matrix proteins (Legakis et al., 2002; Apanasets et al., 2014), it is very likely that conditions chronically disturbing the redox state of the cytosol will affect peroxisome function. Finally, as alterations in peroxisomal ROS production rapidly trigger changes in the mitochondrial balance (Ivashchenko et al., 2011), these organelles may act as upstream initiators of mitochondrial ROS signaling pathways (for more details, see next section).

To which extent peroxisomal β-oxidation influences the cellular or mitochondrial redox state under physiological conditions is not documented. What is interesting to note here is that African green monkey kidney cells (CV-1 cells) (Chu et al., 1995), mouse fibroblasts (LM-tk cells) (Dadras et al., 1998), and rat urothelial cells (MYP3 cells) (Okamoto et al., 1997) overexpressing rat ACOX1 are transformed upon long-term culturing in the presence of fatty acids such as linoleic acid, erucic acid, or nervonic acid. Given that CV-1 cells overexpressing urate oxidase undergo a similar transformation upon exposure to uric acid (Chu et al., 1996), H_2_O_2_ – and not an acyl-CoA – is thought to be the causative factor. However, in the intact animal, the supply of fatty acids to cells will be a limiting factor. For example, in control rats, hepatic H_2_O_2_ production increases when plasma fatty acids levels are higher (Van den Branden et al., 1984). Nevertheless, under basal conditions, hepatic H_2_O_2_ production is comparable in rats and deer mice with or without peroxisome proliferation (and hence altered ACOX1 levels) (Handler et al., 1992).

Clearly, when discussing peroxisomal β-oxidation and ROS, only H_2_O_2_ production is emphasized. However, one should recall that this pathway (as well as any other pathway requiring an activated carboxylate) acts on acyl-CoAs, and not on free fatty acids. Most reviews on the role of the thiol/disulfide redox state in biological systems completely neglect CoA-SH as possible modulator of ROS-mediated signaling events (Hansen et al., 2009). Nevertheless, levels of free CoA-SH are substantial (~10–150 nmol/g tissue), being highest in liver. The majority of this cofactor is confined to the mitochondrial matrix, reaching mM concentrations [e.g., depending on the diet and nutritional status, it was estimated at 3.5–8.5 mM in rat liver (Van Broekhoven et al., 1981; Horie et al., 1986)]. The cytosolic levels are lower, ranging from 0.02 to 0.20 mM (Van Broekhoven et al., 1981; Horie et al., 1986). This indicates that CoA-SH is second in range to glutathione as the most prominent non-protein thiol in mitochondria (for more details, see Antioxidant Systems). Moreover, cellular CoA-SH concentration drops upon treatment with t-butylhydroperoxide and, based on the presence of mixed CoA-glutathione disulfides and their increase during H_2_O_2_ metabolism in perfused rat liver, an interplay between these two thiol compounds exists (Crane et al., 1982). Finally, the level of this cofactor is regulated by complex metabolic pathways, involving more than 25 acyl-CoA synthetases (Watkins et al., 2007), 13 acyl-CoA thioesterases, and 30 acyltransferases, either N-acyltransferases (conjugating enzymes) or O-acyltransferases (acyl-carnitine transferases and other acyltransferases) (Hunt et al., 2005; searches in the HGNC database of human gene names). Hence, addition of β-oxidizable peroxisomal substrates does not only lead to H_2_O_2_ production, but also to a lowering of cellular (cytosolic) CoA-SH. When the CoA-ester is poorly or not degradable, CoA-SH levels can drop significantly. This phenomenon, also described as “CoA sequestration” (Brass, 1994), can occur in metabolic disorders (Mitchell et al., 2008) or upon exposure to xenobiotics and drugs (Brass, 1994). In addition, CoA-esters are chemically reactive species that are known to transacylate the cysteinyl-thiol of GSH, and glutathione depletion has been described upon treatment of cells with certain carboxylates (Grillo, 2011). In conclusion, the CoA-SH/CoA-ester ratio can influence the cellular (mitochondrial) GSH/GSSG balance. This occurs when dealing with xenobiotic carboxylates that can neither be degraded by peroxisomal α- or β-oxidation, nor by mitochondrial β-oxidation. As such, it is very likely that the GSH/GSSG balance is altered in patients with fatty acid oxidation defects.

## Redox signaling between peroxisomes and mitochondria

Over the years, it has become increasingly clear that several cellular processes (e.g., fatty acid oxidation, antiviral signaling, and cell fate decisions) require the proper cooperation between mitochondria and peroxisomes (Dixit et al., 2010; Van Veldhoven, 2010; Fransen et al., 2013; Nordgren and Fransen, 2014; Odendall et al., 2014). This is further evidenced by the observations that both organelles share key proteins of their division machinery (Schrader et al., 2013), and that peroxisomal dysfunction can cause mitochondrial abnormalities (e.g., structural alterations of the inner mitochondrial membrane, reduction in the activities of several RCCs, depletion of mitochondrial DNA, increase in oxidative stress, increase in biogenesis, etc.) (Goldfischer et al., 1973; Baes et al., 1997; Baumgart et al., 2001; Maxwell et al., 2003; Dirkx et al., 2005; Ferrer et al., 2005; López-Erauskin et al., 2013; Peeters et al., 2015; Salpietro et al., 2015). Recently, it was demonstrated that these mitochondrial perturbations closely follow the loss of functional peroxisomes in time (Peeters et al., 2015). However, the molecular mechanisms underlying these changes remain poorly understood. It also remains virtually completely unstudied to what extent mitochondrial damage contributes to peroxisomal dysfunction. In the following subsections, we review and discuss emerging evidence that peroxisomes and mitochondria share an intricate redox-sensitive relationship and cooperate in cell fate decisions. Key issues include possible messengers, mechanisms, and physiological significance.

### Peroxisomes and mitochondria share an intricate redox-sensitive relationship

**Figure 3:**
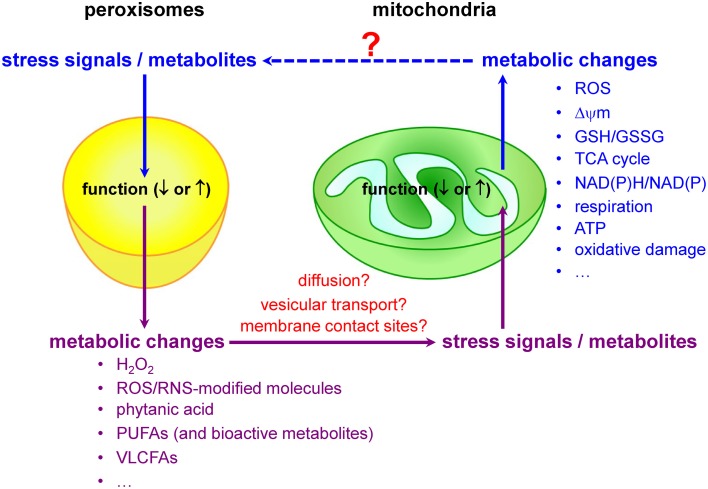
**Peroxisomes and mitochondria share an intricate redox-sensitive relationship**.

Mitochondria and peroxisomes are central organelles in setting cellular redox balance and homeostasis (Noctor et al., 2007; Nordgren and Fransen, 2014). Increasing evidence now also indicates that disturbances and/or deficiencies in peroxisomal lipid and ROS metabolism have, directly or indirectly, an impact on the mitochondrial redox balance (Figure 3). For example, *in cellulo* experiments have shown that inhibition of catalase activity (and hence a concomitant increase in H_2_O_2_ levels) rapidly increases mitochondrial ROS production (Koepke et al., 2008; Ivashchenko et al., 2011; Walton and Pizzitelli, 2012). In addition, it has been observed that catalase, a non-canonical PTS1-containing enzyme (Purdue and Lazarow, 1996), mislocalizes to the cytosol during cellular aging (Legakis et al., 2002) and that this phenomenon precedes the age-dependent decrease in mitochondrial inner membrane potential (Koepke et al., 2007). Importantly, as expression of catalase-SKL, a variant with enhanced peroxisome targeting efficiency, can repolarize mitochondria and reduce the number of senescent cells in late passage cell cultures of human fibroblasts (Koepke et al., 2007), it is reasonable to postulate that peroxisome-derived oxidative imbalance may rapidly impair mitochondrial function (Fransen et al., 2012; Walton and Pizzitelli, 2012). In support of this hypothesis are, among others, the findings that (i) inactivation of ABCD1, a peroxisomal VLCFA transporter causative for X-linked adrenoleukodystrophy (X-ALD), causes oxidative damage to mitochondrial proteins and impairs oxidative phosphorylation (OXPHOS) in the spinal cord of mice (López-Erauskin et al., 2013), (ii) acute and chronic loss of PEX5 function quickly impair the activities of the RCCs I, III, and V in hepatocytes from mice (Peeters et al., 2015), and (iii) the activities of the muscle mitochondrial RCCs II, III, and IV are decreased in a Zellweger syndrome (ZS) patient with homozygous pathogenic mutations in the *PEX16* gene (ZS is the most severe of the peroxisome biogenesis disorders) (Salpietro et al., 2015).

### Redox messengers and modulators

Currently, little is known about the biological messengers that convey redox information between peroxisomes and mitochondria. Potential messengers may include primary ROS/RNS, ROS/RNS-modified molecules, and metabolites. Indeed, it is well known that peroxisomes and mitochondria are actively involved in the metabolism of H_2_O_2_, ^•^NO, and certain lipids that act as signaling molecules (Wang et al., 2014), and—as such—it is very likely that alterations or disturbances in peroxisomal or mitochondrial metabolism may trigger communication events between these organelles. As—to our knowledge—virtually nothing is known about how alterations in mitochondrial activity affect peroxisome function, the next subsections will focus on peroxisomal substrates and metabolites that, upon changes in organelle function, may trigger changes in mitochondrial ROS production due to metabolic stress.

#### Hydrogen peroxide

As (i) peroxisomes contain copious amounts of enzymes that can produce or degrade H_2_O_2_ (see Pro-oxidant Systems and Antioxidant Systems), (ii) peroxisomal H_2_O_2_ can leak into the cytosol (Mueller et al., 2002), and (iii) changes in catalase activity (and hence in peroxisomal H_2_O_2_ metabolism) have a profound impact on mitochondrial redox balance (see Peroxisomes and Mitochondria Share an Intricate Redox-sensitive Relationship) and respiration (Barbosa et al., 2013), it is plausible to suppose that peroxisome-derived H_2_O_2_ can act as a signaling molecule between peroxisomes and mitochondria (Camões et al., 2014). However, the underlying physiological mechanisms are still poorly understood, and it remains to be determined whether peroxisomal H_2_O_2_ exerts its action on mitochondria directly (e.g., via a RIRR response) or indirectly through the activation of non-mitochondrial stress response pathways. In this context, it is interesting to note that inhibition of peroxisomal catalase activity rapidly leads to a decrease in mitochondrial aconitase activity in early-passage human fibroblasts (Walton and Pizzitelli, 2012) and a reduced phosphorylation of CREB1 and PGC1α transcription in skeletal muscle cells (CREB1 is a cAMP response element binding protein that activates the transcription of PGC1α, a transcriptional co-activator critical for mitochondrial biogenesis and function) (Barbosa et al., 2013).

#### Phytanic acid

Phytanic acid is best known for its accumulation in Refsum's disease (plasma levels: ~1.0 mM; normal: <30 μM), a disorder affecting adults and that is clinically characterized by retinitis pigmentosa, peripheral neuropathy, and cerebellar ataxia (Wanders et al., 2011). In addition, the levels of this branched-chain fatty acid are also elevated in patients with rhizomelic chondrodysplasia punctata (RCDP), a more severe disease with cerebellar atrophy caused by death of both Purkinje cells and granular neurons (Powers et al., 1999). Interestingly, loss of these Purkinje cells was postulated to be caused by the incorporation of phytanic acid into cellular membranes, thereby altering intracellular calcium levels and causing mitochondrial dysfunction (Powers et al., 1999). In the meantime, it is known that—when administered to rat hippocampal astrocytes—phytanic acid (50–100 μM) causes a transient rise in cytosolic Ca^2+^, mitochondrial depolarization and ROS generation, and cell death within a few hours of exposure (Reiser et al., 2006; Schönfeld et al., 2006). Phytanic acid (100–500 μM) also enhances the production of O^•−^_2_ in mitochondria isolated from rat brain and heart tissue (Schönfeld et al., 2006; Grings et al., 2012). In these tissues, such treatment also resulted in lower mitochondrial GSH and NAD(P)H levels, a decreased membrane potential, the oxidative modification of both lipids and proteins, and cytochrome c release (Schönfeld et al., 2006; Grings et al., 2012). One concern related to the studies with brain-derived cells is that the phytanic acid concentration used might not be physiological, given that its levels in cerebrospinal fluid are many fold lower than in plasma (<12 nM in controls) (ten Brink et al., 1993). In addition, it has recently been shown that phytanic acid causes Neuro2a cell death via activation of histone deacetylase activity (Nagai, 2015).

#### Pristanic acid

Pristanic acid, the breakdown product of phytanic acid, is further degraded in peroxisomes via β-oxidation. Hence, the plasma level of this 2-methyl-branched fatty acid is increased in patients lacking peroxisomes or with a deficiency in one of the involved β-oxidation enzymes (5-80 μM vs. <3 μM in controls). Based on the clinical phenotype of the latter patients, pristanic acid seems to be linked to adult-onset sensory motor neuropathy as well as visual (retinitis pigmentosa) and intellectual problems. As such, many studies on the toxicity of pristanic acid focus on neurons. Rat astrocytes, oligodendrocytes, and neurons of the hippocampus and cerebellar granule cell layer have been reported to generate more ROS upon exposure to pristanic acid (50–200 μM) (Rönicke et al., 2009; Busanello et al., 2014). Compared to phytanic acid, pristanic acid has a stronger cytotoxic effect on the hippocampal cells: it causes a more profound mitochondrial depolarisation and induces a stronger ROS production (Rönicke et al., 2009). However, whether or not the latter could be due to peroxisomal oxidation was not addressed or investigated in these studies. Note that pristanic acid apparently exerts its toxic effect mainly through its protonophoric action, at least in human skin fibroblasts (Komen et al., 2007). When given to post-nuclear supernatant fractions prepared from rat brain cortex, pristanic acid also causes ROS-generation, as evidenced by decreased GSH levels and increased levels of MDA and protein oxidation (Leipnitz et al., 2011; Busanello et al., 2014). In mitochondrial preparations of rat brain, pristanic acid decreases the ΔΨm and NAD(P)H levels and causes mitochondrial swelling. As the latter process can be prevented by N-acetylcysteine, swelling is most likely caused by ROS-induced damage of the mPTP (Busanello et al., 2012). Note that the *in vitro* effects of pristanic acid can only be observed at rather high concentrations (200 μM).

#### Polyunsaturated fatty acids and their bioactive metabolites

Due to the lack of Δ4-desaturase in mammals, PUFAs containing a double bond at position 4,5 are generated via retroconversion, a process consisting of elongation, Δ6-desaturation, and one β-oxidation cycle, the latter mainly by peroxisomes. This process ensures the formation of important PUFAs like arachidonic (C20:4; ARA) and docosahexaenoic (C22:6; DHA) acid. Once incorporated into membrane phospholipids, PUFAs are main players in the generation of ROS by a non-enzymatic process, called autooxidation. An initial oxidative event, the formation of a hydroperoxy-derivative, will trigger a series of reactions leading, via double bond migration, to the generation of 4-HNE and MDA, better known as thiobarbituric acid-reactive compounds (TBARS) (Gardner, 1989). These lipo-oxidative end-products oxidatively modify proteins. PUFAs are not only important phospholipid constituents, but also the precursors of a large class of bioactive fatty acid derivatives, called eicosanoids (derived from ARA) or docosanoids (derived from DHA). Among the best known are prostanoids (prostaglandins, prostacyclins, thromboxanes), leukotrienes, and ω /ω-1-hydroxy- and epoxy-derivatives, all being classified as signaling lipids. After inactivation, these carboxylates are degraded via peroxisomal β-oxidation (Van Veldhoven, 2010). PUFA-epoxides are oxidative products but, in contrast to TBARS, they are formed enzymatically (Spector and Kim, 2014). PUFA-epoxides are mainly inactivated by the cytosolic EPHX2 (Morisseau and Hammock, 2013). However, due to the presence of a weak PTS1, this enzyme is also targeted to peroxisomes in rat (Arand et al., 1991) and in man (Luo et al., 2008). Given that EPH2 is apparently only found in liver and kidney peroxisomes (Enayetallah et al., 2006), these organelles are unlikely to be important for the hydrolysis of PUFA-epoxides. Nevertheless, the impact of peroxisomes on PUFA levels and metabolism is not only substantial, but also quite complex. For example, in peroxisome biogenesis disorders and some peroxisomal β-oxidation enzyme deficiencies, brain PUFA levels (and especially DHA) are lower (Martinez, 1992). This decrease is seen in all cellular phospholipids, including the mitochondrial ones (Peeters et al., 2015). Additionally, the presence of abnormal very-long-chain PUFAs (generated via a runaway process) has been documented in Zellweger patients (Poulos et al., 1988) and some β-oxidation deficiencies (Infante et al., 2002; Huyhe et al., 2006). Whether or not these PUFA-related changes have particular consequences for ROS-signaling or mitochondrial functioning is not known. PUFA-dependent ROS formation in cultured cells is generally linked to stimulation of plasma membrane-bound NADPH-oxidase. However, in PC12 cells, PUFAs increase the fluorescence intensity of MitoSOXred, indicating mitochondrial ROS production, likely by impairing the electron flux in the respiratory chain (Schönfeld et al., 2011). When given to isolated bovine heart mitochondria, ARA—like other fatty acids—causes uncoupling via inhibition of complex I and III (Cocco et al., 1999). Note that, under these conditions, also more H_2_O_2_ is produced (Cocco et al., 1999).

#### Very-long-chain fatty acids

The accumulation of VLCFAs (C24:0-C30:0) is a biochemical hallmark of X-ALD, a disorder linked to mutations in the peroxisomal ABCD1 membrane transporter and characterized by demyelinisation of the central nervous system. How VLCFAs, mainly found in the cholesterylesters in white matter and adrenals, cause neurodegeneration is not entirely clear, but particular lipids with low abundancy are thought to be involved. Examples include C24:0-lysophosphatidylcholine causing abnormal activation of microglia and apoptosis (Eichler et al., 2008) and VLCFA-gangliosides activating CD8 cytotoxic T-cells via aberrant binding to CD1 (Ito et al., 2001). More recently, oxidative stress has been proposed to contribute to the pathology. This is mainly based on the facts that (i) X-ALD plasma has increased levels of TBARS, carbonyls, and GSSG/GSH ratios (Vargas et al., 2004; Petrillo et al., 2013), (ii) X-ALD red blood cells display increased GPX activity (Vargas et al., 2004), and (iii) cultured X-ALD fibroblasts contain increased levels of modified lysine residues (and especially N1-carboxyethyl-lysine and N1-malondialdehyde-lysine) (Fourcade et al., 2008), elevated catalase and SOD activities (Vargas et al., 2004), and a higher sensitivity to L-buthionine-sulfoximine, an inhibitor of GSH synthesis (Fourcade et al., 2008). Similarly, two-fold more O^•−^_2_ and H_2_O_2_ is produced in ABCD1 (or ACOX1)-silenced 158N murine oligodendrocytes (Baarine et al., 2012b).

Various findings in X-ALD suggest that VLCFAs (or related compounds) affect the mitochondrial compartment. For example, in the dorsal root ganglia of adult X-ALD patients, atrophic neurons were observed with lipidic inclusions in the mitochondria (Powers et al., 2001), and abnormal mitochondria (condensed cristae, myelinoid figures, mitochondrial dissolution) were found in the adrenal cortical cells of (presymptomatic) 12–13 month-old ABCD1-deficient mice (McGuinness et al., 2003). In addition, a defective OXPHOS could be observed in *ex vivo* spinal cord slices from such mice (López-Erauskin et al., 2013), and signs of ROS (e.g., increased MDE-lysine levels) in this tissue could already be demonstrated as early as 3.5 months of age (Fourcade et al., 2008). At 12 months, MDE-lysine levels tended to normalize while markers for carbonylation and glycoxidation increased (Fourcade et al., 2008). Very recently, Reiser and colleagues showed that VLCFAs also diminish mitochondrial Ca^2+^ retention capacity, and that brain mitochondria prepared from 6 month-old *Abcd1* null mice show slightly but significantly higher Ca^2+^ retention capacity than those from corresponding wild-type mice (Kruska et al., 2015). In the context of the OXPHOS observations, it is important to mention that the respiratory chain is apparently normal in mitochondria isolated from skeletal muscle tissue of 9 month-old ABCD1-deficient mice, despite the acculumation of VLCFAs (Oezen et al., 2005). Whether these differences in OXPHOS function represent age-related or sample-specific variations, remains to be investigated. However, here it is of interest to note that (i) the spinal cord is the main X-ALD target tissue (in man) (Berger et al., 2014), (ii) OXPHOS complexes are also severely impaired in hepatocytes isolated from liver-specific *Pex5* null mice (Peeters et al., 2015), and (iii) isolated mitochondria and intact cells may respond differently to an increase in VLCFAs because these lipids also exert detrimental influence on pyridine nucleotide regeneration in the cytosol (Kruska et al., 2015). By treatment of the *Abcd1^−/−^* mice with a mixture of antioxidants (N-acetyl-cysteine, α-lipoic acid, and α-tocopherol), oxidative stress, axonal degeneration, and locomotor impairment were reversed (López-Erauskin et al., 2011). Variable effects of VLCFAs (C24:0, C26:0) on cultured cells have been reported. Most likely, multiple factors (e.g., the concentration, the presence of albumin/serum, the number of cells, and the dissolution and delivery mode of VLCFAs) do contribute to this variation. Unfortunately, these experimental details are rarely documented in detail. Above 20 μM, C24:0, and C26:0 appear to be toxic, and a loss of mitochondrial potential was seen in 158N murine oligodendrocytes, rat C6 glioma cells, rat primary neuronal-glial cells, and rat primary oligodendrocytes (Baarine et al., 2012a). This toxicity was accompanied by an increased production of mitochondrial O^•−^_2_, mitochondrial vacuolization, the destabilization of lysosomes, and a decrease in catalase activity (Baarine et al., 2012a). However, at physiological VLCFA concentrations (1–5 μM, levels found in X-ALD plasma), no effects were seen. Further studies on the 158N oligodendrocytes showed that 20 μM of C24:0 or C26:0 (complexed to cyclodextrin) triggered oxidative stress characterized by overproduction of O^•−^_2_, H_2_O_2_, and ^•^NO associated with lipid peroxidation (e.g., increased levels of 4-HNE, total 7-hydroxycholesterols, and total hydroxyoctadecadienoic acids), protein carbonylation, increased SOD2 activity, and decreased catalase activity and GSH/GSSG ratios (Baarine et al., 2012a). Silencing of the expression of ABCD1 or ACOX1 enhanced the effects of VLCFAs (Baarine et al., 2012a). However, neutral lipid accumulation was only observed with ACOX1 silencing. Human skin fibroblasts appear to be less affected by VLCFAs. Exposure to 100 μM C26:0 did only have minor effects on the inner mitochondrial potential and intracellular ROS production, and only SOD2 was upregulated (Fourcade et al., 2008).

Compared to normal skin fibroblasts, X-ALD fibroblasts are more sensitive to C26:0 in that ROS production starts at lower concentrations (from 10 μM on vs. 50 μM) and GSH levels drop more (Fourcade et al., 2008). Exposure of mouse spinal cord slices to C26:0 (100 μM) resulted in higher expression levels of GPX1 but lower expression levels of SOD1 and SOD2. Related to the cellular studies with VLCFAs, it should be emphasized that C26:0 levels in plasma from healthy controls (<1.5 μM, being the sum of free and esterified C26:0) and X-ALD patients (< 5μM) are extremely low (ten Brink et al., 1993). At physiological VLCFA concentrations (1–5 μM, levels found in X-ALD plasma), no effects were seen in 158N murine oligodendrocytes, rat C6 glioma cells, rat primary neuronal-glial cells, and rat primary oligodendrocytes (Baarine et al., 2012a).

### Mechanisms

A hypothesis gaining prominence is that disturbances in peroxisomal metabolism can trigger redox-related signaling events that ultimately result in increased mitochondrial stress and the activation of mitochondrial stress pathways (Titorenko and Terlecky, 2011; Beach et al., 2012; Fransen et al., 2013). However, the communication pathways involved remain to be established. Potential mechanisms may include (i) the diffusion of signaling molecules from one compartment to the other via the cytosol, (ii) the exchange of molecules via direct membrane contact sites or vesicular transport mechanisms, and (iii) retrograde signaling. Naturally, the communication pathway may differ depending on the identity, reactivity, and selectivity of the messenger. For example, as H_2_O_2_ can diffuse out of peroxisomes (see Hydrogen Peroxide), it is very likely that this molecule can modulate the activity of extra-peroxisomal redox-sensitive proteins (e.g., transcription factors, kinases, and phosphatases) involved in the (transcriptional) control of mitochondrial biogenesis and function. One such example may be AKT1, a serine-threonine protein kinase that positively regulates the activity of CREB1 [see Hydrogen Peroxide and (Barbosa et al., 2013)] and is degraded by the ubiquitin-mediated proteasome pathway in conditions with elevated H_2_O_2_ (Kim et al., 2011). As the biological half-life of some ROS is extremely short (e.g., O^•−^_2_, ~10^−6^s; ^•^OH, ~10^−9^s), it is unlikely that these molecules will be directly transported from one compartment to the other by diffusion or vesicular transport mechanisms (Fransen et al., 2012; and references therein). In this context, it is interesting to note that we recently discovered that the production of excess O^•−^_2_ inside peroxisomes causes cellular lipid peroxidation, and that this in turn triggers a complex network of signaling events eventually resulting in increased mitochondrial H_2_O_2_ production (Wang et al., 2013b). Note that, as (i) there is some evidence that the propagation of ROS signals from the ER to mitochondria is facilitated by membrane contact sites (Verfaillie et al., 2012), and (ii) such contact sites may also exist between peroxisomes and mitochondria (Horner et al., 2011; Schrader et al., 2015), it is possible that these sites are also involved in the redox communication between peroxisomes and mitochondria. Finally, as mitochondria have the ability to generate mitochondria-derived vesicles (MDVs) that selectively transport mitochondrial proteins to either peroxisomes or lysosomes (Neuspiel et al., 2008; Soubannier et al., 2012a), such vesicular transport pathways may also exist for peroxisomes. Here it is interesting to note that, albeit the MDVs destined for the lysosomes are selectively enriched for oxidized proteins, the functional importance of MDV-mediated protein delivery to peroxisomes has not yet been determined (Soubannier et al., 2012b).

### Physiological significance

Currently, it is widely accepted that mitochondrial ROS levels are crucial to regulate the fitness of eukaryotic organisms (Hamanaka and Chandel, 2010). In addition, it is becoming increasingly clear that mitochondria can act as dynamic receivers, integrators, and transmitters of oxidative stress derived from various sources (Nickel et al., 2014). We and others have shown that also disturbances in peroxisomal redox metabolism have an immediate impact on mitochondrial ROS production, both *in cellulo* (Walton and Pizzitelli, 2012; Wang et al., 2013b) and *in vivo* (López-Erauskin et al., 2013; Peeters et al., 2015). In addition, there is strong evidence that defects in peroxisome function as well as excessive ROS-production inside these organelles can trigger mitochondria-mediated cell death (López-Erauskin et al., 2012; Wang et al., 2013b). These and other findings clearly demonstrate that peroxisomal and mitochondrial fitness are closely intertwined, and—as such—it may not come as a surprise that both organelles play a cooperative role in the pathogenesis of at least some neurometabolic diseases. For example, defects in peroxisome biogenesis have been reported to lead to secondary dysfunction of mitochondria, and this may in turn determine—at least in part—the severe phenotype of Zellweger syndrome (Salpietro et al., 2015). The finding that peroxisomes and mitochondria cooperatively function in redox regulation may also offer therapeutic potential for at least some patients with peroxisomal deficiencies. Indeed, as has been shown in preclinical experiments with *Abcd1* null mice, boosting mitochondrial function with pioglitazone (Morató et al., 2013) or activators of SIRT1 (Morató et al., 2015) may normalize redox balance and prevent axonal demise.

## Promises and potential of “Omics” approaches

Oxidative and nitrosative stress-induced modifications are central to a broad range of stress responses, and the accumulation of ROS/RNS can be expected to leave traces of biomarkers at the genome, transcriptome, proteome, and metabolome levels (Aebersold, 2003; Ma et al., 2013). As state-of-the-art “-omics” technologies allow detection of subtle biological variations, such platforms offer unique opportunities for researchers to study the molecular effects of oxidative stress at system level. The goal of this section is to provide the reader with a few examples of how data mining of publicly-available large-scale data may be used to gain additional insights into potential redox signaling pathways between peroxisomes and mitochondria (we do not intend to give an exhaustive overview).

A first example is RedoxDB, a curated database of experimentally-verified protein oxidative modifications (http://biocomputer.bio.cuhk.edu.hk/RedoxDB/) (Sun et al., 2012). Searching this database revealed that multiple peroxisomal proteins in mammalian cells [e.g., catalase, 3-ketoacyl-CoA thiolase (ACAA1), and hydroxyacid oxidase 2 (HAO2)] contain cysteine thiol groups that are susceptible to oxidation by ROS/RNS and can undergo S-nitrosylation (RSNO), S-sulfenylation (RSOH), and/or S-thiolation (RSSG) (Doulias et al., 2013). Note that ACAA1 catalyzes the final step in the oxidation of straight-chain acyl-CoAs, and that HAO2 is an FMN-dependent enzyme that oxidizes long-chain L-2-hydroxy acids to ketoacids at the expense of O_2_ with concomitant production of H_2_O_2_. The physiological relevance of these observations remains to be determined. However, as (i) these modifications are likely to impact enzyme activity and stability (Stolz et al., 2002; Ortega-Galisteo et al., 2012; Gould et al., 2013), and (ii) alterations in peroxisomal fatty acid β-oxidation and H_2_O_2_ metabolism have been shown to affect mitochondrial function (see Peroxisomes and Mitochondria Share an Intricate Redox-sensitive Relationship and Redox Messengers and Modulators), such reversible cysteine modifications may participate in signaling processes between mitochondria and peroxisomes.

The second example is AGEMAP (Atlas of Gene Expression in Mouse Aging Project), a gene expression database that catalogs changes in gene expression in mice as a function of age (Zahn et al., 2007). The idea behind this database is that the identities of age-related genes provide important clues about mechanisms (e.g., stress response) that drive transcriptional changes in old age (e.g., oxidative stress). Upon profiling the effects of aging on gene expression in different tissues dissected from mice of ages 1, 6, 16, and 24 months and comparing these data with DNA microarray data on aging from human muscle, Becker and colleagues discovered that 17 genesets are commonly age-regulated in multiple human and mouse tissues (Zahn et al., 2007). The most relevant ones in the context of this manuscript are the genesets associated with peroxisomes and the mitochondrial electron transport chain, both of which show an overall decrease in expression with age (for comparison, the genesets associated with lysosomes and the inflammatory response showed a common increasing trend in expression with age).

A third and last example concerns the proteomic analysis of a subdomain of the ER, called the mitochondrial-associated ER membrane (MAM). Previous confocal microscopy studies have shown that MAM also physically connects mitochondria to peroxisomes during antiviral response (Horner et al., 2011). In addition, activation of the antiviral innate immune response has been reported to alter peroxisomal and mitochondrial morphology (Dixit et al., 2010; Horner et al., 2011). Evidence that mitochondria and peroxisomes physically interact, can also be inferred from proteomic datasets. Indeed, proteomic analysis of MAM fractions isolated from mouse brain (Poston et al., 2013), cytomegalovirus-infected human fibroblasts (Zhang et al., 2011), and SenV- or hepatitis C virus-infected Huh7 human hepatoma cells (Horner et al., 2015) have demonstrated that these fractions also contain peroxisomal matrix (e.g., AGPS, ACOX1, CAT, etc.) and membrane (e.g., ABCD1, PEX11β, PEX14, etc.) proteins. Interestingly, some of these proteins (e.g., CAT and PEX14) are differentially enriched in MAM fractions from infected and non-infected cells. Although the biological significance of these observations remains to be established, it has been hypothesized that the changes in expression of individual proteins may reflect changes in organelle interactions during the antiviral signaling response and the generation of new signaling sites through these organelle interactions (Horner et al., 2015). Importantly, as the MAM compartment represents a hot spot for the intracellular signaling of important pathways, including phospholipid synthesis and ROS generation and activity (Giorgi et al., 2015), it is tempting to speculate that this compartment also provides an axis through which stress stimuli and metabolites can be transmitted from peroxisomes to mitochondria (see Mechanisms). However, experimental evidence to support this hypothesis is currently lacking.

## Concluding remarks

Peroxisomes and mitochondria are pivotal team players in cellular redox metabolism. In addition, growing evidence suggests that mitochondria can act as dynamic receivers, integrators, and transmitters of peroxisome-derived mediators of oxidative stress, and that alterations in the peroxisomal redox state are likely to impact mitochondrial redox activity. Therefore, in order to understand how a decline in peroxisome function can be associated with cellular aging and the initiation and progression of oxidative stress-related diseases, it is critical to gain more insight into the molecular mechanisms by which peroxisomes and mitochondria communicate. Future studies should focus on (i) the link between peroxisomal/mitochondrial (dys)function and cellular redox balance, (ii) the identification of the proximal targets of peroxisome-derived ROS/RNS, (iii) the molecular mechanisms underlying the redox communication between peroxisomes and mitochondria, and (iv) the validation of novel targets and mechanisms in primary cells and tissues from patients and mice suffering from peroxisomal or oxidative stress-related disorders. The outcome of such studies may open up exciting new avenues for the community of researchers working on cellular responses to organelle-derived oxidative stress, a research field in which the role of peroxisomes is currently highly underestimated and an issue of discussion.

### Conflict of interest statement

The authors declare that the research was conducted in the absence of any commercial or financial relationships that could be construed as a potential conflict of interest.

## Abbreviations

ACOX: acyl-CoA oxidase
ARA: arachidonic acid
DHA: docosahexaenoic acid
EPHX: epoxide hydrolase
ETC: electron transport chain
4-HNE: 4-hydroxy-2-nonenal
GLRX: glutaredoxin
GPX: glutathione peroxidase
GSH: reduced glutathione
GSR: oxidized glutathione reductase
GSSG: oxidized glutathione
MAM: mitochondria-associated membrane
MDA: malondialdehyde
mPTP: mitochondrial permeability transition pore
NOS: nitric oxide synthase
NOX: NADPH oxidase
OXPHOS: oxidative phosphorylation
PUFA: polyunsaturated fatty acid
PRDX: peroxiredoxin
RCC: respiratory chain complex
RIRR: ROS-induced ROS release
RNS: reactive nitrogen species
ROS: reactive oxygen species
SOD: superoxide dismutase
TRX: thioredoxin
TSC: tuberous sclerosis complex
TXNRD: thioredoxin reductase
VLCFA: very-long-chain fatty acid
X-ALD: X-linked adrenoleukodystrophy
XDH: xanthine dehydrogenase.
